# First person – Stanley ‘Michii’ Kanai

**DOI:** 10.1242/dmm.049553

**Published:** 2022-04-29

## Abstract

First Person is a series of interviews with the first authors of a selection of papers published in Disease Models & Mechanisms, helping early-career researchers promote themselves alongside their papers. Stanley ‘Michii’ Kanai is first author on ‘ Auriculocondylar syndrome 2 results from the dominant-negative action of PLCB4 variants’, published in DMM. He is a postdoctoral fellow in the lab of Dr David Clouthier at Department of Craniofacial Biology, University of Colorado Anschutz Medical Campus, Aurora, CO, USA, investigating cell-signalling mechanisms in craniofacial development and disorders.

**How would you explain the main findings of your paper to non-scientific family and friends?**

Craniofacial defects in newborns are one of the leading causes of infant mortality and can have life-long consequences for affected individuals. The overall goal of our research is to understand the causes of facial birth defects and to explore potential treatment strategies to prevent or mitigate these defects. In this study, we investigated auriculocondylar syndrome 2 (ARCND2), a rare disorder characterized by lower jaw defects, fusion of jaw joint bones and misshapen ear lobes (referred to as question mark ear). ARCND2 is associated with gene variations in *PLCB4* that produce mutated versions of the phospholipase C β4 (PLCB4) protein, although it was not clear if and how they cause ARCND2 in humans. PLCB4 normally acts as a relay that converts signals from outside the cell to a response inside the cell, but we show that this process is disrupted by mutant PLCB4 in a dominant-negative manner in cell culture experiments. Based on these results, we hypothesize that mutant PLCB4 is likely to prevent cells from forming correct facial bones and cartilages during embryonic development. Our experiments show that introducing a human *PLCB4* gene variant into mice results in facial defects similar to those seen in individuals with ARCND2. In addition, we found a new bone containing tooth-like structures in the skull of *Plcb4* mutant mice, and then compared with human anatomy to find these structures in the skull of an individual with ARCND2. The tooth-like structure resembles a bone present in the skull of reptiles, suggesting that disrupting PLCB4 can re-activate ancient programs governing facial development, resulting in facial birth defects.

**Figure:**
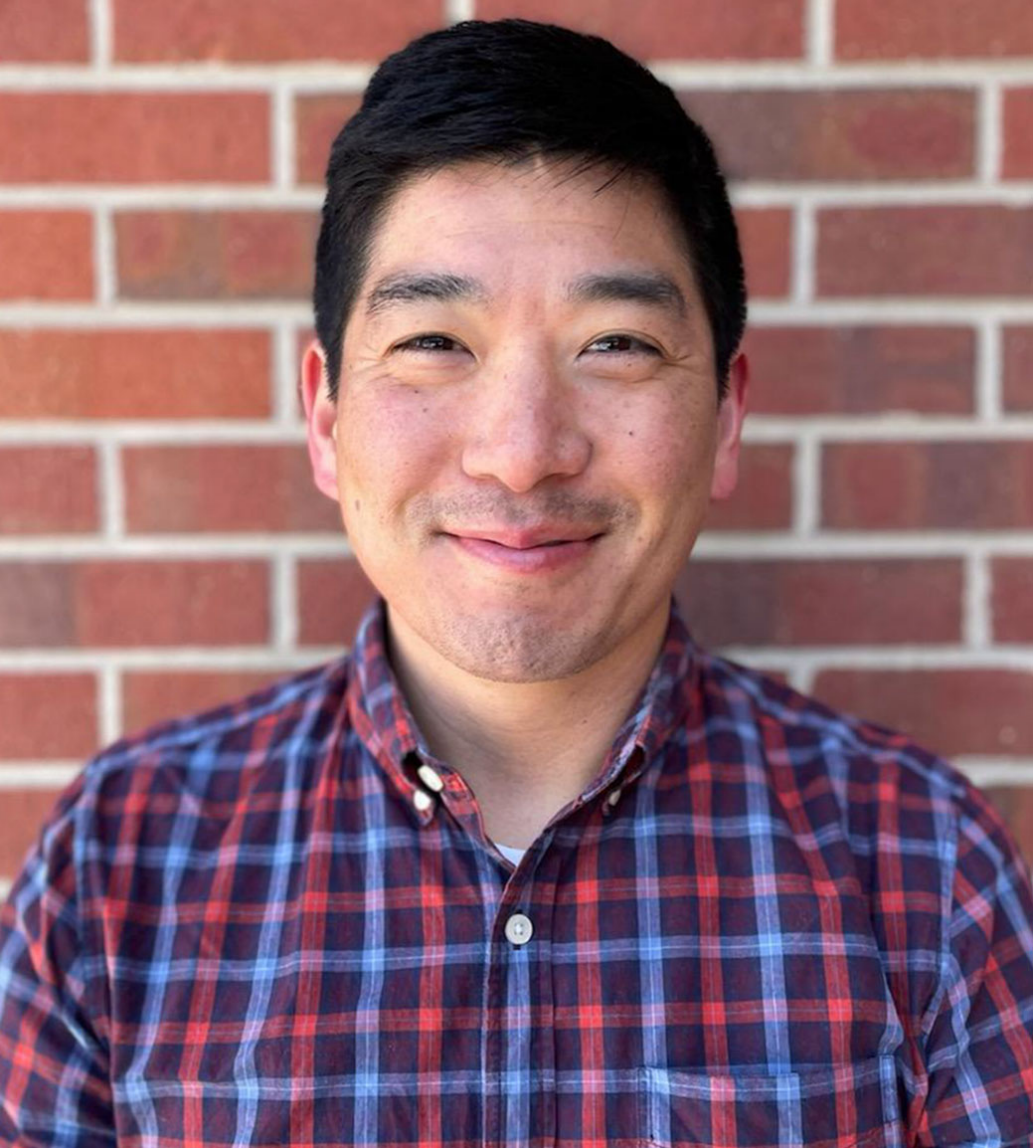
Stanley ‘Michii’ Kanai

**What are the potential implications of these results for your field of research?**

These results lay the groundwork for investigating other puzzling aspects of ARCND2. For example, there is considerable phenotypic variation in individuals with ARCND2, both within family members and between families, suggesting that additional factors influence the severity of craniofacial malformations. By establishing PLCB4 variants as a causative factor for ARCND2, we and others can begin to explore whether environmental or genetic factors modify disease severity. These results may also have implications for personalized medicine. By establishing the role of a specific signal transduction pathway in ARCND2, we now have an opportunity to target this pathway, pharmacologically or otherwise, to prevent craniofacial anomalies. My hope is that our results will motivate future studies to determine the feasibility of this approach. Finally, our findings raise interesting questions about the plasticity of evolutionary adaptations; it would be exciting to see whether minor modifications in other signalling factors can also re-activate ancient developmental programs.

“By establishing the role of a specific signal transduction pathway in ARCND2, we now have an opportunity to target this pathway, pharmacologically or otherwise, to prevent craniofacial anomalies.”

**What are the main advantages and drawbacks of the model system you have used as it relates to the disease you are investigating?**

We began our study using a mammalian cell culture model to characterize the functional consequence of PLCB4 variants. These results provided a strong rationale to extend our investigation into a mouse model. The main advantage of the mouse model is that the developmental processes and anatomy of the craniofacial skeleton in mice are highly conserved in humans. Therefore, craniofacial anomalies in mutant mice likely reflect the disease state in humans. The drawbacks, however, are that generating mouse models of disease is slower and more expensive relative to other model systems. We circumvented these issues by generating an F0 mouse model for ARCND2, using CRISPR/Cas9 to knock in a disease allele into the *PLCB4* locus. Although F0 animals are mosaic, our results indicate that the ARCND2 gene variants are pathogenic and provides motivation to invest additional time and resources to generate a stable mouse line for future studies. Overall, the tandem use of cell culture assays and an F0 mouse model offers a relatively rapid and rigorous approach towards establishing the genetic and cellular basis of a disease.

**Figure:**
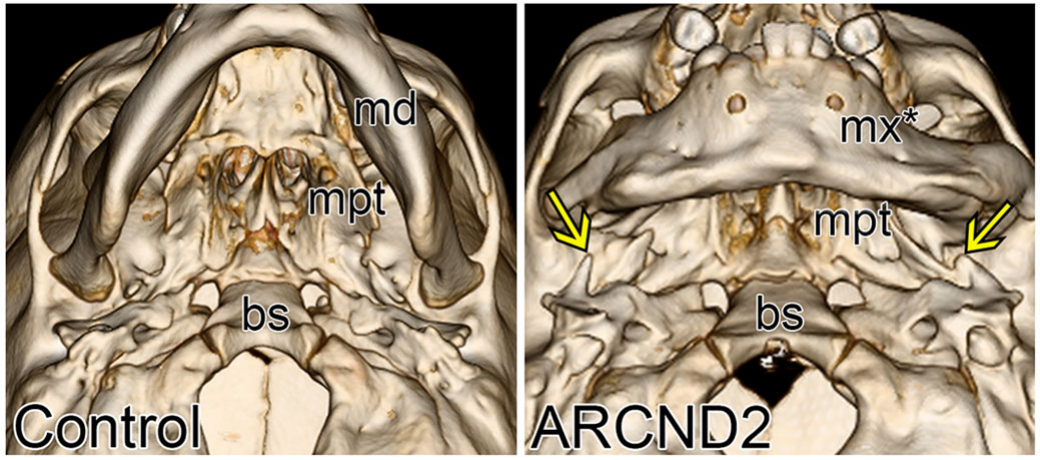
**Three-dimensional renderings of computed tomography (CT) scans from a control individual (left) and an individual with ARCND2 (right), showing the inferior surface of the skull.** Ectopic tooth-like structures (arrow) were found, with the help of the ARCND2 mouse model. bs, basisphenoid; md, mandible; mpt, medial pterygoid bone; mx*, transformed mandible.

**What has surprised you the most while conducting your research?**

I was surprised that all PLCB4 variants examined in this study behaved in a dominant-negative manner. Although our structural modelling predicted that the missense mutations in the catalytic pocket of PLCB4 would result in some kind of functional consequence, why these mutations cause dominant-negative interference rather than loss of function is not clear. I was also shocked to find that the ectopic bone structures in our mouse model were also present in human individuals with ARCND2. This was another reminder of the power of the mouse model for studying craniofacial development.

**Describe what you think is the most significant challenge impacting your research at this time and how will this be addressed over the next 10 years?**

I see two major challenges in our research. The first challenge is to understand the functional consequences of disease-associated gene variants. Advances in sequencing technology have made it possible to identify genetic variants associated with common and rare diseases at an unprecedented rate, although determining the functional significance and causal role of these variants is lagging. A coordinated effort between clinical geneticists and basic scientists is needed to accelerate the pace of variant validation using *in vitro* and *in vivo* approaches. The second challenge is in understanding how disease variants, like those studied here, affect cell signalling pathways *in vivo*. Engineering genetically encoded signalling sensors that work in vertebrate model organisms is not trivial, as *in vivo* sensors often require greater selectivity and sensitivity than those used in cell culture models to account for the complexity of a living organism. Fortunately, many different sophisticated signalling sensors are being developed for *in vitro* use, with multiple groups also establishing reporter lines in zebrafish to detect acute signalling events such as calcium flux and MAP kinase activity. Over the next 10 years, I hope to build on these innovations and develop a research program that investigates the mechanism of human disease variants using *in vitro* assays, novel *in vivo* signalling reporters, and model organisms.

**What changes do you think could improve the professional lives of early-career scientists?**

The academic research career-path is challenging, but having institutional support specifically for postdocs makes a big difference. For example, my institution, the University of Colorado Anschutz Medical Campus, has a dedicated office that provides guidance for long-term career planning, grant-writing workshops and resources for wellness management.

**What's next for you?**

In addition to mice, I am using zebrafish to study mechanisms of craniofacial development and disorders. I am currently generating zebrafish models for several craniofacial disorders and zebrafish lines that express fluorescent signalling reporters, including the one used in this study, so that we can monitor signalling events in real time during normal and abnormal development.
